# Cotrel-dubousset instrumentation for the correction of adolescent idiopathic scoliosis. Long-term results with an unexpected high revision rate

**DOI:** 10.1186/1748-7161-7-13

**Published:** 2012-06-18

**Authors:** Franz J Mueller, Herbert Gluch

**Affiliations:** 1 Department of Spinal Surgery and Scoliosis Centre, Behandlungszentrum Vogtareuth, Germany; 2 Hospital for orthopedic and trauma surgery, Hospital Barmherzige Brueder, Regensburg, 93042, Germany

**Keywords:** Adolescent idiopathic scoliosis, Surgery, Outcome

## Abstract

**Background:**

For many years, the CD instrumentation has been regarded as the standard device for the surgical correction of adolescent idiopathic scoliosis (AIS). Nevertheless, scientific long-term results on this procedure are rare. Therefore, we conducted a retrospective follow-up study of patients treated for AIS with CD instrumentation and spondylodesis.

**Methods:**

A total of 40 patients with AIS underwent CD instrumentation in our department within 3 years and between 1990 and 1992. For the retrospective analysis, first all the patient documents were reviewed, and pre-/postoperative X-ray images as well as those at the latest follow-up were analysed. Furthermore, it was attempted to conduct a clinical survey using the SRS-24 questionnaire, which was sent to the patients after a preceding announcement on the phone.

**Results:**

Radiologically, the frontal main curvature was improved from a preoperative angle of 69.2° to a postoperative angle of 35.4°, and the secondary curvature was improved from a preoperative angle of 42.6° to a postoperative angle of 20.5°. The latest radiological follow-up at average 57.4 months post surgery showed an average loss of correction of 9.6° (main curvature) and 4.6° (secondary curvature), respectively.

Within the first 30 days post surgery, 3 out of 40 patients (7.5%) received early operative revision for the dislocation of hooks or rods.

At an average of 45.7 months (range 11 to 142 months), 19 out of 40 patients (47.5%; including 2 patients with early revision) received late operative revisions: The reasons were late infection (10 out of 40 patients; 25%) with the development of fistulae (7 cases) or putrid secretion (3 cases), which was resolved with the complete removal of instrumentation after all. The average time until revision was 35.5 months (range 14 to 56 months) after CD instrumentation. Furthermore, complete implant removal was necessary in 8 out of 40 patients (20%) for late operate site pain (LOSP). The average time until removal of instrumentation was 62.7 months (range 18 to 146 months) post surgery; and one patient received partial device removal for prominent instrumentation 11 months post surgery. Altogether, only 22 out of 40 CD instrumentations (55%) were still in situ.

After an average period of 14.3 years post surgery, it was possible to follow-up 14 out of 40 patients (35%) using the SRS-24 questionnaire. The average score was 93 points, without showing significant differences between patients with or without their instrumentation *in situ*.

**Conclusions:**

Retrospectively, we documented for the first time a very high revisions rate in patients with AIS and treated by CD instrumentation. Nearly half of the instrumentation had to be removed due to late infection and LOSP. The reasons for the high rate of late infections with or without fistulae and for LOSP were analysed and discussed in detail.

## Introduction

For the surgical treatment of adolescent idiopathic scoliosis (AIS), various anterior and posterior procedures and instrumentations were developed. With regard to posterior procedures, two different well-known instrumentations must be distinguished:

With the development of the Harrington rod [1], a long-segment rod and hook instrumentation was available for the first time, and became the worldwide standard procedure for many years in the surgical treatment of AIS. According to a retrospective meta-analysis, Harrington rods were used in more than 85% of such cases between 1958 and 1993 [2]. With this instrumentation, frontal correction was achieved primarily by distraction and elongation of the concave side, and by the compression of the convex side of the curve. The disadvantage of this method was the flattening of the spinal profile [3]. Moreover, there have been reports of high revision rates with the complete removal of the instrumentation due to chronic complaints of the lumbar spine [4]. The instrumentation has also required postoperative, external stabilisation for approx. 6 to 12 months with a plaster cast or an orthesis in order to avoid a loss of initial curve correction [1,5-7].

With the development and introduction of a novel double-rod instrumentation by Cotrel and Dubousset about 25 years later [8], the disadvantages of the Harrington rod seemed to be eliminated. Early results with a small number of patients showed that the CD instrumentation did not only provide better lateral and frontal curve correction, but also a significant correction of vertebral rotation and thus a marked reduction in the cosmetic rib hump deformity [9]. Moreover, the instrumentation gives greater primary stability so that postoperative management with an external orthesis was no longer required. On the other hand, long-term results are rare in the literature and, therefore, we conducted a retrospective long-term follow-up study including patients treated for AIS with CD instrumentation and spondylodesis at our centre.

## Materials and methods

**Figure 1:**
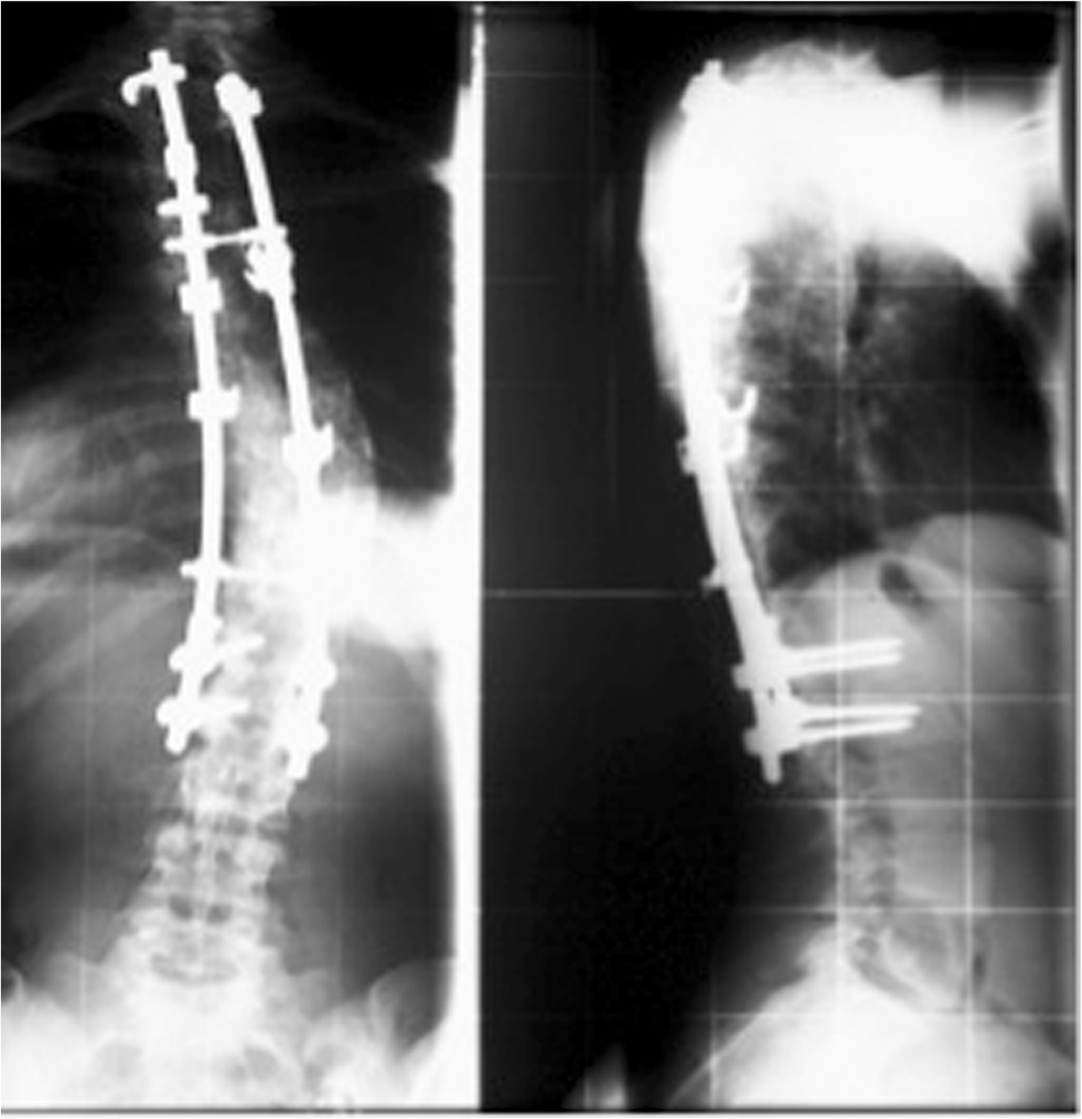
X-rays of CD instrumentation with hooks and pedicle screws (so-called hybrid technique).

Between March 1990 and September 1992, a total of 42 consecutive patients with AIS underwent surgical correction with CD instrumentation (Sofamor; Figure 1) by four different surgeons. The CD is a double rod system made of steel, which allows segmental fixation through lamina hooks and/or conical pedicle screws. During this period, no other posterior procedure was employed for the surgical treatment of AIS. Before this period, we used Harrington rods [1] or Luque instrumentation [10] for this indication, while after the period, only an instrumentation made by titanium alloy was used [11]. The indication for surgery was the progression of the scoliosis and/or a main curve of more than 45° in the frontal plane. The exclusion criteria were spinal abnormalities, e.g. wedge-shaped vertebra, or a previous operative procedure such as an Ascani rod or spondylodesis for anterior correction. 2 out of 42 patients had spondylolysis of the fifth lumbar spine vertebra, with the fixation of CD instrumentation including sacral levels; therefore, we excluded these 2 patients. Anterior release to mobilise a rigid main curve was not an exclusion criterion and was done in 3 out of 40 patients (7.5%) for a severe or rigid main curve.

Additional procedures or measures such as rib osteotomy or thoracoplasty were not performed in any of the 40 patients.

For the preparation and mobilisation prior to the operation, all of the patients carried out Cotrel self-extension over a period of 2 to 3 weeks, though a halo extension was not applied to any patient. Immediately preoperatively, and if necessary intraoperatively, all the patients were given intravenous cephalosporin as a prophylactic antibiotic. The surgical procedure, after the exposure of the spine, involved the mobilisation of the scoliosis initially by resection of the spinous processes, decortication of the laminae, facet joint cleaning, and division of the ligamentum flavum on the concave side of the curvature. This was followed by the correction of the scoliosis by inserting the hooks and pedicle screws with the loading of the two anatomically shaped vertical rods with rotation *in situ* with additional compression or distraction of the segments as needed. No one was instrumented or fused caudally until up to the sacrum and only 2 out of 40 patients to the fifth lumbar vertebra. Two transverse connectors were placed cranially and caudally between the two vertical rods in all of the patients. A wake-up test was performed to check the intraoperative neurology after the insertion of the vertical rod on the concave side; neurophysiological monitoring was not employed. For spondylodesis only local bone material reduced to chips was employed. All patients received autologous blood with or without cell saver, and no patient received blood products that were not autologous. All patients were mobilised in principle from the second or third postoperative day without a corset.

For the study, the demographic data of all 40 patients were initially recorded from the medical files: age at the time of surgery and sex, operation time, blood loss, and documented complications. All pre- and postoperative and the latest follow-up spinal radiographs (average 57.4 months post surgery) in both planes (with the patients standing on cassettes that were 36 inches long) were then analysed with regard to the following parameters: curve patterns were classified by the method based on King-Moe [12] and Scoliosis research society terminology; measurement of the frontal main and secondary curves using the Cobb method [13], number and height of the fused vertebra and number of hooks and pedicle screws employed. Frontal balance, determined on the basis of the horizontal distance, in millimetres, from the centre of the C7 vertebral body to the centre of the sacrum. Imbalance was defined as a horizontal distance of > 20 mm. Apical vertebral translation was defined as the distance, in millimetres, between the plumb line and the mid-portion of the vertebral body at the apex of the curve. The sagittal thoracic kyphosis angle was measured between T5 and T12 and the lordosis angle between L1 and S1, also using Cobb‘s method [13]. Because of the relatively low accuracy of the measurements, the radiological measurement of vertebral rotation or clinical measurement of ribcage projection was omitted. CT scans were not performed routinely, neither pre- nor postoperatively.

At the time of the empirical data collection, after an average period of 14.3 years (range 189 to 159 months) post surgery, it was attempted to contact the patients via telephone in order to conduct a clinical evaluation using the SRS-24 questionnaire. Due to various reasons (e.g. contact not possible, projection), it was only possible to assess 14 out of 40 patients (35%).

### Statistics

For statistical analysis, SPSS Version 8 software for Windows was used for the statistical analysis, and P values of ≤ 0.05 were considered significant. Descriptive statistics were used to determine the means, standard deviations (SD), and ranges. Comparisons between the variables were performed using the Student’s t test and the Kruskal-Wallis test.

## Results

### Demographic data

The demographic data included 40 patients (28 female, 12 male), with an average age at the time of surgery of 16.0 years (range 13 to 21 years). According to the SRS terminology there were 23 right thoracic, 10 double major curves, 4 thoracolumbar, and 3 left thoracic curves. According to the King-Moe classification [12], 17 patients had type II curves; 7 patients type III, and 8 patients type IV curves. Posterior fusion with the CD instrumentation was performed in all cases. The average blood loss was 2770 ml (range 1500 to 5500 ml), and the average operation time was 325 min (range 225 to 410 min). Fixation with the CD instrumentation included 13.4 vertebrae (range 8 to 16) on average. Except for the first 3 consecutive cases, for which only hooks were used, the two rods were fixed by the hybrid technique, a combination of hooks and pedicle screws, with an average of 8 hooks and 4 screws per patient. A total of 325 hooks and 161 pedicle screws were inserted in 40 patients. The pedicle screws were inserted mainly in the lumbar region of the instrumentation. 24 patients (60%) were instrumented caudally until lumbar level L3, and 16 patients (40%) were instrumented until lumbar level L4 or L5.

### Radiographic results

In the frontal plane, the mean preoperative primary curve was 69.2° (range 50° to 100°). Postoperatively, the primary curve was 35.4° (range 13° to 70°), giving a correction rate of 48.8%. At the most recent follow-up evaluation, the Cobb angle of the curve was 41.6°, giving a final correction rate of 39.9%. The mean preoperative secondary curve was 42.6° (range 21° to 73°). Postoperatively, the secondary curve was 20.5° (range 2° to 46°), giving a correction rate of 51.9%. At the most recent follow-up evaluation, the Cobb angle of the curve was 25.1°, giving a final correction rate of 41.1%. The frontal balance, as determined by the mediolateral offset of the C7 vertebral body centre in relation to CSVL, was 14.6 mm on average, postoperatively, and 3 out of 40 patients (7.5%) had frontal imbalance of more than 20 mm offset (range 25 to 36 mm), postoperatively. Apical translation (apex of primary curve to CSVL) measured 63.5 mm pre-, 30.9 mm postoperatively and 37.3 mm at the final follow-up, giving a final correction rate of 41.3%. In the sagittal plane, the average preoperative thoracic kyphosis angle (T5 to T12) curve was 22.4° (range 2° to 63°). Postoperatively, the thoracic kyphosis angle measured 23.6°, only one patient had thoracic hyperkyphosis of more than 40°. Moreover, the average preoperative lumbar lordosis angle (L1 to S1) curve was 57.3° (range 30° to 87°). Postoperatively, the lumbar lordosis angle measured 54.9° (range 30° to 78°).

### Complications

There was no direct or indirect operative mortality. Furthermore, there was no permanent neurological complication. In one case, surgical correction was stopped incompletely because of massive bleeding, and then the correction was successfully concluded 14 days later.

According to the medical files, 21 out of 40 patients (52.5%) received one or more operative revisions, for a total of 23 surgical revisions:

Within the first 30 days post surgery, 3 out of 40 patients (7.5%) received early operative revision for the dislocation of hooks or rods.

At an average of 45.7 months (range 11 to 142 months), 19 out of 40 patients (47.5%; including 2 patients with early revision) received late operative revisions.

At this occasion, we documented the revision rate of the highest significance (9 out of 16 procedures; 56%), not in the first year of introduction but rather for 1992, the third and last year of application. Despite thorough data analysis, we could not find any explanations for this.

The reasons were late infection (10 out of 40 patients; 25%) with the development of fistulae (7 cases) or putrid secretion (3 cases), which was resolved with the complete removal of instrumentation after all. We documented 5 out of 7 fistulae at the distal end of the instrumentation. The average time until revision was 35.5 months (range 14 to 56 months) after CD instrumentation. There were no bacteriological findings of any pathogens after a maximum time of cultivation of 48 hours.

Furthermore, complete implant removal was necessary in 8 out of 40 patients (20%) for late operate site pain (LOSP). No infections or non-unions were detected intraoperatively, but there was a partial implant loosening in the caudal section of the implant in 6 cases, including corrosion in 2 out of 6 cases and broken cranial transverse connectors in 2 other cases. The average time until removal of instrumentation was 62.7 months (range 18 to 146 months) postoperatively.

Moreover, one more patient received partial device removal for prominent instrumentation 11 months postoperatively.

Therefore, only 22 out of 40 CD instrumentations (55%) were still in situ.

The statistical analysis showed only tendency but no significant influence for the distal end of the instrumentation: 8 out of 16 patients (50%) with instrumentation including lumbar level 4 or 5 received implant removal for infection or LOSP. On the other side, 10 out of 24 patients (41.7%) with distal end of instrumentation until lumbar level 1, 2 or 3 received implant removal. This difference was not statistically significant (p > 0.05). Moreover, the statistical analysis showed no significant influences for the removal of instrumentation compared to patients with instrumentation still in situ (18 versus 22 patients) for e.g. sex, type of curve (right thoracic, double or other curves), or other demographic parameters.

### Results for SRS-24

Only 14 out of 40 patients (35%) completed the SRS-24 questionnaire after a mean of 14.3 years postoperatively. In the SRS-24 questionnaire, the total score averaged 93.3 points out of a maximum 120 points (min. 71 to max. 106 points) at the follow-up. The analysis of the questionnaire showed no significant differences between the 5 patients with instrumentation still *in situ* (average 96.4 points) and the 9 patients after the removal of the instrumentation (average 91.5 points).

## Discussion

The retrospective study (Evidence Level 4) was primarily aiming at assessing the clinical and radiological long-term results of AIS patients after dorsal CD instrumentation. For this, it was initially possible to include all of the 40 consecutive patients who had undergone surgical procedures in our spine department during a period of 3 years. Compared to other so-called monocentric studies, this number of cases seems somewhat small in total; however, it may be considered sufficient when taking into account the follow-up period [14-16]. Surprisingly, the analysis of the patient records already showed a high number of surgical revisions that had not been documented before in the literature [14-22]. As a consequence, empirical data collection was designed in a way to confirm reasons or influencing factors for this as a secondary objective.

Therefore, a more detailed discussion is required:

First of all, it must be emphasised that all surgical procedures had been conducted following strict and standardised aseptic criteria in a modern operation room with a laminar flow section, and that single-shot antibiotic prophylaxis had been given and repeated, respectively, if required. Furthermore, our previously published data from 1993 to 1996 had demonstrated clearly better results with regard to infection rates [11]. Moreover, only autologous bone substance had been used for spondylodesis, and no allogeneic blood had been given. Empirical data analysis showed consistent data and results when compared to other studies with regard to patient age, scoliosis classification, and preoperative and immediately postoperative curvature/corrections [14].

After an average postoperative period of 57.4 months, the latest x-ray images showed a minor loss of correction – of both main and secondary curvatures – that was similar to the results demonstrated in other study [14].

However, compared to other studies [14-22], empirical data analysis showed a clearly increased length of instrumentation: an average of 13.4 segments had been included in the instrumentation. Therefore, it is likely that the increased spinal stiffness over a longer distance may be the reason for the high rate of late revisions (also considering that the implant design had a high profile), particularly when considering that most of the fistulae (5 out of 7) as well as partial implant loosening in cases of LOSP had mainly been seen in the distal instrumentation section. As a result, we consider the length of instrumentation as a possible factor influencing the complication and revisions rate. However we found only a weak tendency but not significant influence in cases where the distal instrumentation included L4 or L5 when compared to cases where distal instrumentation included only L1, L2 or L3 (50% versus 41.7%).

It must be emphasised from a historical point of view that, prior to the introduction of CD instrumentation, the surgeons had been trained in the use of the Harrington rod for many years (and its distal hooks reached down to the lower lumbar spine) – however, this system is not consistent with the philosophy and usage of the CD. Nevertheless, no “learning curve” with significantly increased complication rates has been seen during the first year of introduction. In contrast, the highest revision rate (9 out of 16 procedures; 56%) was seen in 1992, which was the third and last year of application. Despite thorough data analysis, we could not find any explanations for this.

Over time, 40 procedures were performed by 4 different surgeons, which is a basic deviation when compared to other studies [14,17,19], because this complex surgery has mainly/exclusively been conducted by senior authors. Statistical analyses did not show significant differences between surgeons with regard to revision rates, and a multivariate analysis did not show other influencing factors such as the duration of surgery, or blood loss. On the other hand, we documented a clearly prolonged duration of operation time and higher volumes of blood loss due to longer instrumentation distances and the positioning of hooks and pedicle screws when compared to most of the studies with lower complication rates [14,17]. Both factors are known to be inversely proportional to the risk for complications, such as infections. In fact, this could have been at least one factor contributing to the revision rates due to late infections, since these have been shown to be commonly caused by so-called dermal pathogens [23,24]. *Propionibacterium acnes* is a specific pathogen; however, detection requires a particular agar and a incubation period of up to 14 days. This might be an explanation for the fact that this pathogen was not identified in any of our cases with clinically manifest late infections, since all the specimens had been incubated for a routine period of only up to 48 hours at the time of surgery. Furthermore, proprietary unpublished study results show that the intraoperative detection of *Propionibacterium acnes* in the surgical wound significantly increases with the duration of surgery.

In our study group, we had 8 patients with back pain without clinical evidence of pseudarthrosis or manifest infections at the time of surgical revision/complete implant removal. Cook et al. [19] documented a total of 6 operative revisions due to LOSP in 49 patients (12%). They defined LOSP exclusively as midline and parascapular pain without a clinically apparent cause. The cause of LOSP in these patients was not clear, but pseudarthrosis was ruled out. Moreover, the authors believed that LOSP is most likely caused by the local soft tissue reaction to the implant. Another possibility is the implant prominence, which may cause soft tissue irritation and back pain. Kostuik [25] reported 5 out of 49 patients (10%) with CD implants who required implant removal. On the other side, LOSP was not distinguished clearly from late deep infection and there are overlaps [26]. All of our LOSP patients have been shown to have intraoperative pathological findings: There was a partial implant loosening in the caudal section of the implant in 6 cases, including corrosion in 2 out of 6 cases, and broken cranial transverse connectors in 2 other cases. Therefore, in contrast to Cook et al. [19], we postulate that LOSP is always based on pathological causes, unless the contrary has been proved. Thus, strictly speaking, LOSP represents a general term for different pathological findings (e.g. corrosion, bursitis, implant loosening, low virulent infection), which may become manifest intraoperatively only at the time of revision.

After an average period of 14 years post surgery, it was not possible to encourage more patients to participate in a clinical follow-up, despite intense effort. However, this is in accordance with other clinical results after long-term follow-up [7]. There were multiple reasons for this, with a change of residence or no interest in follow-up being the most common ones. Therefore, it was possible to follow-up on only 14 out of 40 patients using the SRS-24 questionnaire, which was sent to the patients after initial telephone contact. As a consequence, it is not possible to draw a generally applicable clinical conclusion, if only 35% of the patient population were followed-up on. However, the analysis of the SRS-24 data showed a trend that both patients with CD and patients without implants *in situ* mainly had a good quality of life post surgery. We finally summarised the clinical results of other studies investigating CD instrumentation in Table 1.

**Table 1:** The table represented the overall summaries by the different authors for CD instrumentation in the treatment for AIS

**A**	**B**	**C**	**D**	**E**	**F**
2007	Boss	38	7	satisfaction, functional status and subjective cosmetic improvement was high	none
2007	Bjerkreim	86	10	Scores for EuroQol were within the normal range	45% had back pain within the last year
2006	Weigert	41	>2	SRS-24: fair or better in all domains	reoperation rate was 21.6%
2003	Helenius	57	13	SRS: 97 points	11% reported back pain often or very often
2003	Bago	110	5	-	reoperation rate was 21%
2000	Cook	49	9	-	reoperation rate was 24%
1998	Lenke	76	6	outcome was favourable	38% reported occasional pain in the spine
1997	Takahashi	30	6	the overall clinical results were satisfactory	20% prevalence of low back pain

A: year of publication.

B: author’s name.

C: number of patients.

D: mean of follow up in years.

E: positive conclusions.

F: negative conclusions.

## Conclusion

Retrospectively, we documented for the first time a very high revisions rate in patients with AIS and treated by CD instrumentation. This high rate of surgical revisions has not yet been documented in the literature. Nearly half of the instrumentation had to be removed due to late infection and LOSP. The reasons for the high rate of late infections with or without fistulae and for LOSP were analysed and discussed in detail.

## Competing interests

The authors declare that they have no competing interests.

## Authors' contributions

FJM has contributes in conception and design and acquisition of data, analysis and interpretation of data, drafting the manuscript and revising it critically; HG has contributed in conception and design of data, drafting the manuscript and given the final approval of manuscript. Both authors read and approved the final manuscript.
